# The Polysaccharides from the Aerial Parts of *Bupleurum chinens*e DC Attenuate Epilepsy-Like Behavior through Oxidative Stress Signaling Pathways

**DOI:** 10.1155/2022/7907814

**Published:** 2022-04-07

**Authors:** Xiaomao Li, Yan Liu, Siyi Wang, Yikai Jiang, Adnan Mohammed Algradi, Yuanyuan Zhou, Wei Guan, Juan Pan, Haixue Kuang, Bingyou Yang

**Affiliations:** Key Laboratory of Basic and Application Research of Beiyao (Heilongjiang University of Chinese Medicine), Ministry of Education, Heilongjiang University of Chinese Medicine, 24 Heping Road, Xiangfang District, Harbin 150040, China

## Abstract

*Bupleurum chinense* DC. is a traditional Chinese medicine with a long medicinal history and is often used as the main ingredient in prescription drugs for epilepsy. The aerial parts of *B. chinense* DC. have similar efficacy and composition to *B. chinense* DC. Therefore, we speculated that the aerial parts of *B. chinense* DC. could be used in the treatment of epilepsy. Polysaccharides from the aerial parts of *B. chinense* DC. were selected to explore their therapeutic effects on epilepsy and their potential mechanism of action. The study is aimed at clarifying the antiepileptic effects of the polysaccharides from the aerial parts of *B. chinense* DC. and their potential underlying mechanisms. The chemical profile of the aerial parts of *B. chinense* DC. polysaccharides (ABP) was characterized by FT-IR spectrum and HPLC chromatogram. To determine the therapeutic effects of ABPs on epilepsy, we established a kainic acid- (KA-) induced rat model of epilepsy, and through H&E staining, Nissl staining, immunohistochemistry, biochemical analysis, ELISA, and Western blot analysis, we explored the mechanisms underlying the therapeutic effects of ABPs on epilepsy. The monosaccharide content of ABP included galacturonic acid (45.19%), galactose (36.63%), arabinose rhamnose (12.13%), and mannose (6.05%). Moreover, the average molecular weight of ABP was 1.38 × 10^3^ kDa. ABP could improve hippocampal injuries and neuronal function in the KA-induced epilepsy rat model. ABP significantly inhibited oxidative stress in the hippocampus of KA-induced rats. More importantly, ABP could regulate TREM2 activation in the PI3K/Akt/GSK-3*β* pathway to inhibit neuronal apoptosis, including increasing the expression of superoxide dismutase and lactate dehydrogenase and decreasing the expression of malondialdehyde. The current study defined the potential role of ABP in inhibiting the development of epilepsy, indicating that ABP could upregulate TREM2 to alleviate neuronal apoptosis, by activating the PI3K/Akt/GSK-3*β* pathway and oxidative stress in epilepsy.

## 1. Introduction

Epilepsy is one of the most common neurological diseases. Currently, there are approximately 50 million people with epilepsy worldwide, accounting for 1% of the total number of patients with any disease worldwide [1]. The prevalence of epilepsy in China is approximately 7%, with 300,000-400,000 new patients diagnosed every year. In the treatment of epilepsy, the prescribed drugs show a poor treatment effect in approximately 30% of patients, which leads to refractory epilepsy [2]. Repeated and long-term seizures tend to lead to the activation of inflammatory factors, calcium homeostasis and increased free radical generation, and selective brain damage of neurons in the hippocampus, including neuronal necrosis and apoptosis [3]. The brain is one of the most sensitive organs to oxidative stress. As it mostly obtains energy through the aerobic metabolism of the mitochondrial respiratory chain, it produces a large number of free radicals. The brain is rich in unsaturated fatty acids and has a weak antioxidant capacity, which makes it very vulnerable to oxidative stress injury [4]. The high sensitivity of the brain to oxidative stress injury suggests that oxidative stress plays an important role in the pathophysiology of epilepsy. In recent years, many molecular biology and genetic studies have attempted to clarify the relationship between oxidative stress and epilepsy.

Polysaccharides are biopolymers that are usually the main bioactive component of traditional Chinese medicine and the affective component of dietary supplements. Recent studies have shown that polysaccharides have important antioxidative, antitumor, anti-inflammatory, and antifatigue effects and play a relevant role in immune regulation [5–9]. At present, many natural polysaccharides are considered safe and effective therapeutic agents for epilepsy because they are nontoxic [10]. Thus, these polysaccharides have attracted considerable interest in the development of nontoxic natural foods and drugs. *Bupleurum chinense* DC. is a traditional Chinese medicine with a long medicinal history and extensive clinical application because of its antiepileptic and sedative-hypnotic effects [10]. Its main active ingredients are polysaccharides and saponins. The aerial part of *Bupleurum chinense* DC. was rich in resources, and its composition and pharmacological action were similar to that of *Bupleurum chinense* DC. [11]. The *B. chinense* DC. polysaccharides showed a strong oxidation capacity and possessed promising hepatoprotective effects against GalN-induced liver damage, which might be mediated through augmentation of antioxidant defenses [12]. So, the aerial part of *Bupleurum chinense* DC. polysaccharides also might possess the oxidation capacity. And, the preliminary study of our group showed that the extracts from the aerial parts of *B. chinense* DC. had a good therapeutic effect on epilepsy through behavioral evaluation [13]. However, the effects of the polysaccharides from the aerial parts of *B. chinense* DC. on epilepsy have rarely been reported. Therefore, in this study, we aimed to explore the potential mechanisms of the polysaccharides from the aerial parts of *B. chinense* DC. for the treatment of epilepsy by inhibiting oxidative stress.

## 2. Materials and Methods

### 2.1. Plant Materials and Reagents

The aerial parts of *B. chinense* DC. were collected in Daqing, Heilongjiang Province, China, in 2017 and identified as *B. chinense* DC. by Professor Rui-feng Fan from the Heilongjiang University of Chinese Medicine. The aerial parts were kept in the laboratory of Chinese Medicine Chemistry of the Heilongjiang University of Chinese Medicine (specimen number 20170910). Kainic acid (KA) was obtained from Sigma-Aldrich (Shanghai, China), a sodium valproate oral solution was obtained from Sanofi (Hangzhou, China), and Dian Xian Ning tablets were purchased from Kunming Chinese Medicine Factory Co., Ltd. (Kunming, China). All other analytical reagents were purchased from Sigma-Aldrich.

### 2.2. Animals

Four-to-six-week-old male Sprague-Dawley (SD) rats (*n* = 70) weighing 180-200 g were purchased from Liaoning Changsheng Biotechnology Co., Ltd. (SCXK (Liao) 2020-0001). The experimental animals were raised under standardized conditions at 20°C-23°C and with a humidity of 50%-60%. All experiments were approved by the Animal Experiment Committee of the Heilongjiang University of Chinese Medicine (20201230001).

### 2.3. ABP Extraction

The weight of the aerial parts of *B. chinense* DC. was 1.0 kg. The petroleum ether extract was degreased three times for 12 h each, and the residue of the volatilized dry petroleum ether was heated three times during reflux extraction with distilled water at 100°C for 3 h each. The extract was then combined, filtered, and concentrated to 1/4 of the original volume, and then, anhydrous ethanol was added to adjust the volume to obtain an ethanol volume fraction of 70%. After centrifugation at 4,000 × g for 8 min, the precipitate was collected to obtain the crude polysaccharides from the above-ground aerial parts of *B. chinense* DC. The protein in the crude polysaccharide was removed using the Sevag reagent (chloroform : n − butanol = 4 : 1, *V*/*V*) and repeated four times. The supernatant was collected and dialyzed with distilled water using a 3500 Da dialysis bag for 72 h. ABP was obtained after freeze-drying.

### 2.4. Characterisation of the ABP

5 mg of the ABP was placed in an agate mortar, an appropriate amount of dry KBr powder was added, ground, and mixed, and the infrared spectrum was determined after tablet pressing, using a scanning range of 4,000-400 cm^−1^ [14–16]. The monosaccharide composition of the ABP was assessed using reverse-phase HPLC, as described previously [17, 18]. We dissolved 10 mg of ABP samples in 2 mL of 2 M trifluoroacetic acid. The monosaccharides from hydrolyzed ABP were analyzed using Sugar-Pak TM1 chromatographic column (6.5 × 300 mm) by using HPLC.

### 2.5. Experimental Design

After anesthetizing 60 rats, their lateral ventricle was stereo-positioned, and a custom-made microinjection tube was used to lower a 3.8 mm needle into the right lateral ventricle to inject a uniform injection volume of 3 *μ*L of a KA solution at a concentration of 0.5 *μ*g/*μ*L (50 rats) or to inject 3 *μ*L of normal saline (10 rats). After the injection, the needle was retained for 5 min to establish a KA-induced epilepsy rat model [19]. Drug administration was initiated the day after successful model establishment.

Ten unoperated male SD rats as the control group (CON). 10 male SD rats inject normal saline as the sham-operated group (Sham). 50 male SD rats with epilepsy were randomly divided into five groups with 10 rats in each group: model group (MOD), western medicine positive drug group (sodium valproate oral solution (VPA), 180 mg/kg b.w., po., 28 days), traditional Chinese medicine positive drug group (Dianxianning tablets (DXN), 500 mg/kg b.w., po., 28 days), ABP high-dose group (ABPH, 200 mg/kg b.w., po., 28 days), and ABP low-dose group (ABPL, 50 mg/kg b.w., po., 28 days). And CON, sham, and MOD groups were given the same amount of normal saline (po., 28 days).

### 2.6. Behavioural Assessment

Epileptic seizures were observed and recorded in rats after the cerebral injection of KA. The epileptic seizure grade was assessed according to the Racine grading criteria: grade 0: no reaction, no seizure, and no difference in behavioral performance from that of normal rats; grade I: facial clonus, including blinking, whisker shaking, rhythmical chewing, and wet dog head shaking; grade II: grade I and rhythmic nodding or shaking of the head; grade III: grade II and paroxysmal clonus of forelimb; grade IV: grade III and hind limb standing; grade V: grade IV, ankylosis, and spasm of both hind limbs, falling backward or sideways, losing balance, and violent convulsions of the limbs [20].

### 2.7. Haematoxylin and Eosin Staining

The rats were anesthetized via abdominal injection of 1% pentobarbital sodium (50 mg/kg), intubated through the ascending aorta, irrigated with normal saline, infused with 500 mL of 4% paraformaldehyde, and immersed in 4% paraformaldehyde after brain extraction for 4-6 h. Each brain was immersed in a 30% sucrose solution, and a frozen slicer was used to cut 30 mm-thick slices in the coronal position. These slices were then attached to the glass-bearing slices, defatted in water, soaked with hematoxylin for 10 min, and washed briefly with water. They were then differentiated under a microscope with a 1% hydrochloric acid-alcohol solution, rinsed with water for 30 min, soaked with eosin for 5 min, dehydrated to make them transparent, and sealed [21]. The CA1 and CA3 regions of the hippocampus were selected for observation.

### 2.8. Nissl Staining

The brain sections were degreased with dimethyl benzene and an alcohol gradient, soaked in distilled water for 5 min, soaked with 1% toluidine blue at 60°C for 40 min, rinsed with distilled water for 5 min, differentiated with 95% alcohol for 1 min, dehydrated with anhydrous alcohol to make them transparent, and sealed. The CA1 and CA3 regions of the hippocampus were then observed under a light microscope (Nikon Eclipse Ci-L, Japan, magnification, ×200) [22].

### 2.9. Immunohistochemistry

Brain slices were immersed in 0.1 M phosphate buffer saline (PBS) containing 0.5% Triton X-100 for 60 min and a 0.6% H_2_O_2_ ethanol saline solution (50% ethanol and 0.9% sodium chloride) for 45 min. Normal sheep serum was added to them, followed by incubation for 60 min. The residual serum was poured out without washing the slices, and these were dripped with rabbit-derived glial fibrillary acidic protein polyclonal antibody (Abclonal, Wuhan, China, 1 : 500) and incubated at 4°C for 36-48 h. Biotinylated secondary antibody (sheep anti-rabbit) was added to the slices dropwise, and these were incubated at 25°C for 2 h. Streptavidin labeled with horseradish enzyme was added dropwise, followed by incubation at room temperature for 2 h. Between each step, the slices were washed three times with 0.01 M PBS for 10 min each. The sections were then stained with 3, 3′-diaminobenzidine tetrahydrochloride, counterstained with hematoxylin, dehydrated to make them transparent, and sealed. The positive expression of the hippocampus CA1 and CA3 regions were observed under a microscope, and the *f* positive expression was calculated using Image J 18.0 (200x magnification).

### 2.10. Biochemical Analysis

Total protein was extracted from the fresh hippocampal tissues of the experimental rats of each group. The malondialdehyde (MDA) content and the superoxide dismutase (SOD) and lactate dehydrogenase (LDH) activities were determined using UV spectrophotometry according to the manufacturer's instructions for each test kit (Nanjing Jiancheng Bioengineering Institute, Nanjing, China). The expression of Caspase 3, COX2, TLR4, HMBG1, NMDAR 1, GABA AR, GAD65, GAD67, and GAFP was determined using specific methods, according to the instructions of the corresponding ELISA kits (Shanghai Enzyme-linked Biotechnology Co., Ltd.).

### 2.11. Western Blot Analysis

Total protein was extracted from the fresh hippocampal tissues of the rats of each group. The total protein of 20 *μ*g of each sample was separated using polyacrylamide gel electrophoresis, which was then transferred onto a polyvinyl difluoride membrane. After sealing, the protein was incubated with primary antibodies against Bax, Bcl-2, caspase 3, TREM2, PI3K, p-Akt, Akt, and GSK-3*β* (1 : 1000, Abclonal, Wuhan, China) at 4°C overnight. The ECL reagent was added, followed by incubation at room temperature for 1 h with an appropriate dilution of the corresponding HRP-labelled secondary antibody (Abclonal). GAPDH (1 : 1000, Abclonal) was used as an internal reference. The expression level results were compared with those of each control group, which was used as the standard, and with the results of other groups.

### 2.12. Cell Culture

The highly differentiated PC12 rat pheochromocytoma cells (Cell Bank of Chinese Academy of Sciences, Shanghai) were cultured in RPMI 1640 containing 10% fetal bovine serum and placed in a 5% CO_2_ incubator at 37°C for routine passage. The confluent cells (70%-80% confluency) were digested using trypsin and washed with PBS, and a single-cell suspension was prepared, which was then used to inoculate 96-well plates at a density of 5000 cells/well. After 24 h of culture, the cells were randomly divided into six groups after cell adherence: (1) control group: normal culture; (2) model group: RPMI 1640 with H_2_O_2_ (500 *μ*M) for 4 h, and then replaced with RPMI 1640 containing 10% fetal bovine serum for 24 h; (3) VPA group: RPMI 1640 containing valproic acid sodium (1 mM), which was replaced after 4 h with 500 *μ*M H_2_O_2_; (4) ABP groups: RPMI 1640 for 4 h, which was then replaced with RPMI 1640 containing ABP at different concentrations (20, 40, and 80 *μ*g/mL).

### 2.13. Cell Viability Assay

After treatment, the culture medium was removed, the cells were washed with PBS, and 100 *μ*L of RPMI 1640 and 10 *μ*L of a CCK8 solution were added to each well. After a slight shock, the cells were incubated at 37°C for 1 h. Then, the absorbance of each well was measured at 450 nm, and the relative cell viability was calculated.

### 2.14. Apoptosis Assays Using Flow Cytometry Analysis

Confluent PC12 cells were used to inoculate 6-well plates at a density of 1 × 10^6^ cells/well and then divided into groups for posttreatment. Then, the cells were gently scraped and washed twice with PBS. A total of 1 × 10^5^ cells were collected from each group, and the supernatant was discarded after 4°C 1000 r/min centrifugation 15 min. Next, 195 *μ*L of an Annexin V-FITC staining solution and 5 *μ*L of a PI staining solution were added, and the cells were incubated in a 37°C incubator for 20 min without light after a slight shock. The cells were washed once with PBS and resuspended. Fluorescence detection was performed using flow cytometry.

### 2.15. Statistical Analysis

Statistical analyses were conducted using SPSS 17 (SPSS Inc., Chicago, IL, USA). All data are presented as the mean ± standard error (SEM). Statistical significance was analyzed using two-way analysis of variance (ANOVA), and multiple comparisons between groups were made using the Tukey test. Statistical significance was set at *P* < 0.05.

## 3. Results

### 3.1. The Characterisation of ABP

**Figure 1:**
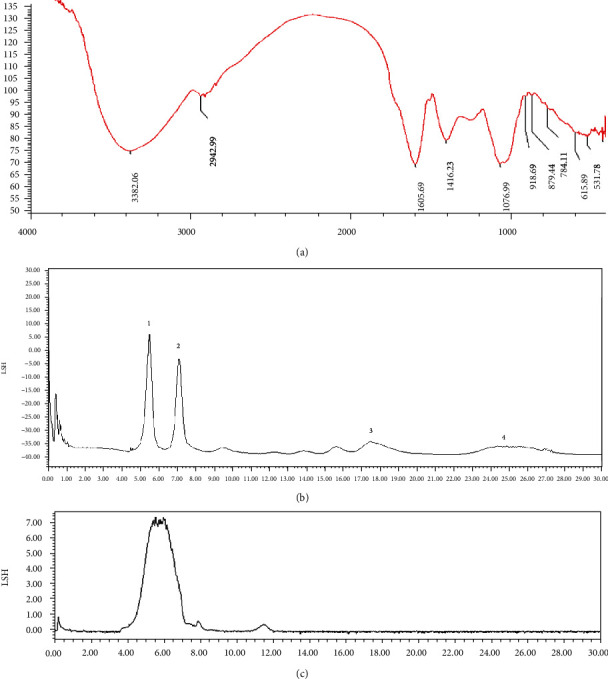
The characterization of ABP. (a) The infrared spectrum of the ABP. (b) Monosaccharide composition of ABP on HPLC. (c) The average molecular weight of ABP.

As shown in Figure 1(a), there is a wide and smooth strong absorption peak near 3,400 cm^−1^ (3,382.05 cm^−1^), which is the O-H stretching vibration peak, and the absorption was caused by the intramolecular and intermolecular O-H stretching vibration of sugar. A very weak characteristic absorption peak was observed at 2942.99 cm^−1^, namely, the absorption peak corresponding to the C-H stretching vibration [22, 23]. The absorption peaks in these areas are characteristic absorption peaks of sugars. The absorption peak at approximately 1,615 cm^−1^ (1,605.69 cm^−1^) is the stretching vibration peak of C=O, and the weak absorption peak at 1420 cm^−1^ (1416.23 cm^−1^) is the in-plane bending vibration peak of C-H [24]. There are two furanose absorption peaks in the 1100-1010 cm^−1^ range, which indicate that ABP contained furanose. However, a weak characteristic absorption peak was observed around 890 cm^−1^, confirming the presence of *β*-glycosidic bonds linking the sugar residues [25].

The monosaccharide composition of the ABP was analyzed using HPLC after hydrolysis, and the ABP contained galacturonic acid (45.19%), galactose (36.63%), arabinose rhamnose (12.13%), and mannose (6.05%) (Figure 1(b)). Furthermore, the average molecular weight of ABP was 1.38 × 10^3^ kDa (Figure 1(c)).

### 3.2. Antiepileptic Effects of ABP on the KA-Induced Rat Model of Epilepsy

**Figure 2:**
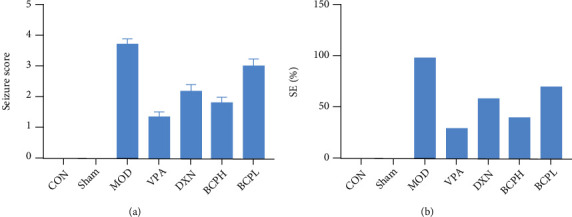
Antiepileptic effects of ABP on KA-induced rats. (a) Seizure core of epilepsy rats after treatment; (b) SE% of epilepsy rats after treatment. Compared with CON and sham groups, the difference was significant (^#^*P* < 0.05); compared with the MOD group, the difference was significant (^∗^*P* < 0.05).

The rats in the sham group had no specific behavior or seizures after the injection of normal saline into their hippocampus. After the KA injection, the rats in the MOD group had seizures of varying degrees, with an incidence of 100%, and 80% of the rats showed simultaneous rhythmic convulsions of the limbs (grade IV or above). The epileptic seizure degree and the behavioral score of rats in the ABPH group were significantly lower than those of the rats in the MOD group (*P* < 0.05) (Figure 2).

### 3.3. Histological and Immunohistochemical Analyses

**Figure 3:**
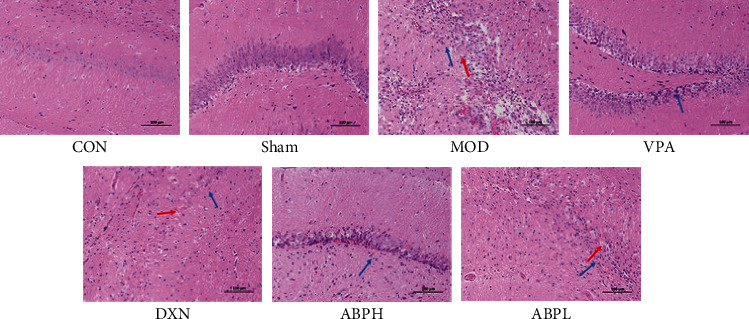
The HE staining of KA-induced rats with treatment.

**Figure 4:**
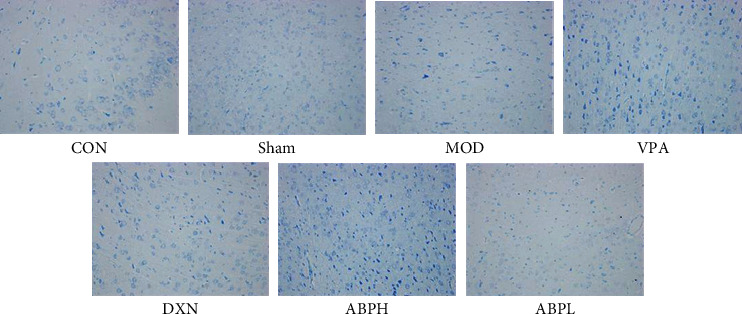
The Nissl staining of KA-induced rats with treatment.

The neurons in the CON and sham groups were neatly arranged, with clear cell outlines, abundant cytoplasm, basophilic, and had around and centered nucleus and clear nucleoli. In the MOD group, the neuron boundaries were not clear, the cell body was wrinkled, cells had a reduced volume, having lost the typical polygon shape, and became round or triangular, the cytoplasm was vacuolated or concentrated and dark, the nucleus was concentrated and deeply stained, and no nucleoli could be observed. The voids around the neurons increased, and there were more holes left after the disappearance of the neurons. The degree of degeneration and necrosis of the hippocampal neurons and the number of neurons affected by these in the VPA and ABPH groups were lower than those in the MOD group. Neuronal injury was improved, and the pyramidal cell nucleus hyperchromatism, fragmentation, or dissolution was reduced in the VPA and ABPH groups, but no significant improvement was observed in the ABPL group (Figures 3 and 4).

**Figure 5:**
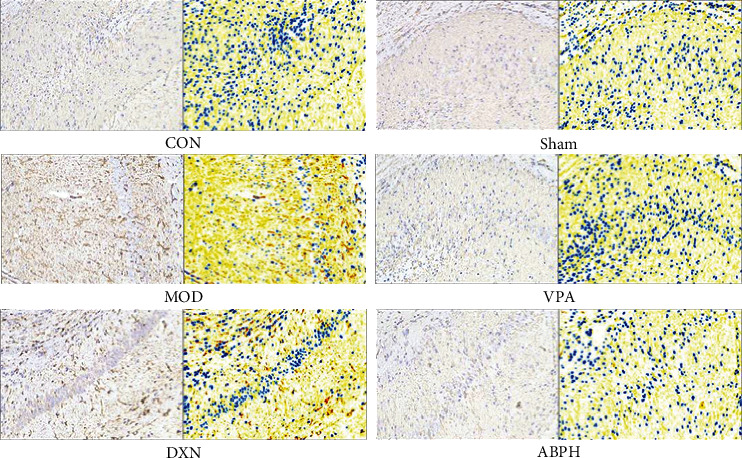
GFAP positive cell expression of KA-induced rats with treatment.

Three sections from each animal were randomly selected to observe the number of GFAP-positive cells in the hippocampus. The results showed that the number of GFAP-positive cells in the hippocampus of the MOD group rats was significantly higher than that of the CON and sham group rats (*P* < 0.01). The number of GFAP-positive cells of the positive drug and ABPH groups was significantly lower than that of the MOD group (*P* < 0.01), while there was no significant difference between the CON and sham groups regarding this parameter, as shown in Figure 5.

### 3.4. Oxidative Stress Index Analysis

**Figure 6:**
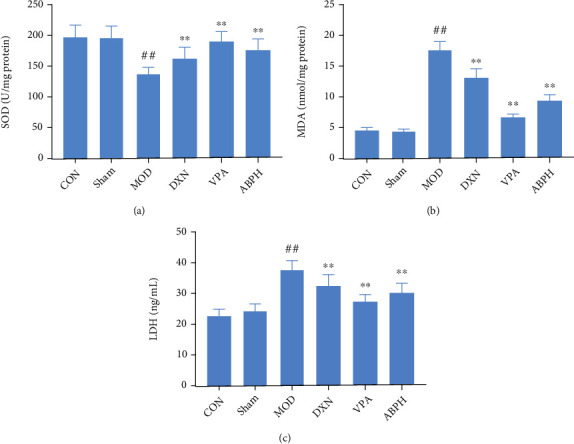
The oxidative stress index levels of KA-induced rats with treatment. (a) The content of SOD in KA-induced epilepsy rats with treatment; (b) the content of MDA in KA-induced epilepsy rats with treatment; (c) the content of LDH in KA-induced epilepsy rats with treatment. Compared with CON and sham groups, the difference was significant (^#^*P* < 0.05); Compared with the MOD group, the difference was significant (^∗^*P* < 0.05).

Compared with those in the CON and sham groups, the content of MDA and the activity of LDH in the MOD group were significantly higher, while the activity of SOD was significantly lower (*P* < 0.01). Compared with those in the MOD group, the activity of SOD was significantly higher and the content of MDA and the activity of LDH were significantly lower in the ABPH group (*P* < 0.01), as shown in Figure 6.

### 3.5. Neurotransmitter Content Analysis

**Figure 7:**
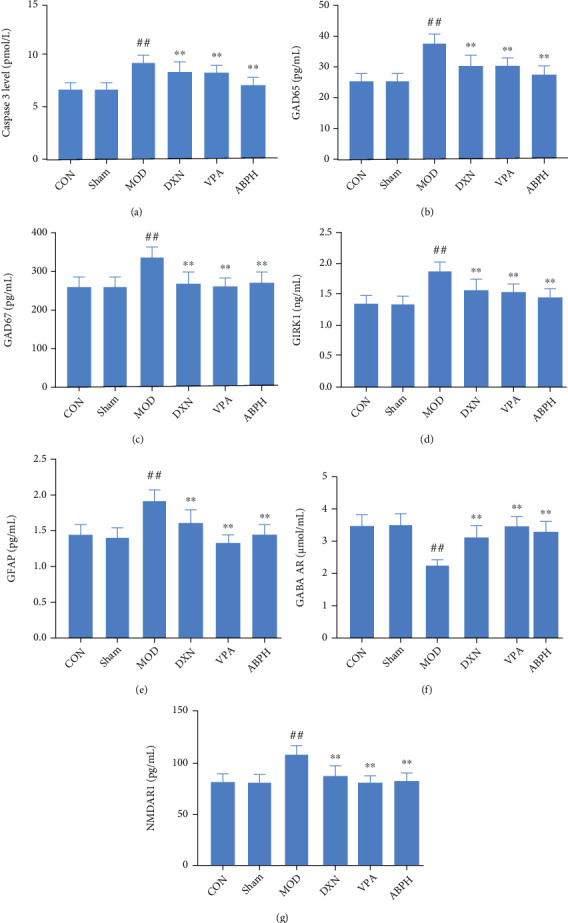
The apoptotic factors and neurotransmitters levels of KA-induced rats with treatment. (a) The content of caspase 3 in KA-induced epilepsy rats with treatment; (b) the content of GAD65 in KA-induced epilepsy rats with treatment; (c) the content of GAD67 in KA-induced epilepsy rats with treatment; (d) the content of GIRK 1 in KA-induced epilepsy rats with treatment; (e) the content of GFAP in KA-induced epilepsy rats with treatment; (f) the content of GABA AR in KA-induced epilepsy rats with treatment; (g) the content of NMDAR 1 in KA-induced epilepsy rats with treatment. Compared with CON and sham groups, the difference was significant (^#^*P* < 0.05); compared with the MOD group, the difference was significant (^∗^*P* < 0.05).

The effects of ABP on the neurotransmitter content of the KA-induced rats were explored. As shown in Figure 7, the caspase 3, GAD65, GAD67, GIRK1, GFAP, and NMDAR1 contents were significantly higher (*P* < 0 01) in the MOD group rats than in the CON group rats, while GABA AR content was significantly lower (*P* < 0.01). Interestingly, the caspase 3, GAD65, GAD67, GIRK1, GFAP, and NMDAR1 contents were significantly lower, and GABA AR content was significantly higher in the ABPH group rats than in the MOD group rats (*P* < 0 01).

### 3.6. Regulation of the Oxidative Stress Pathways in KA-Induced Rats

**Figure 8:**
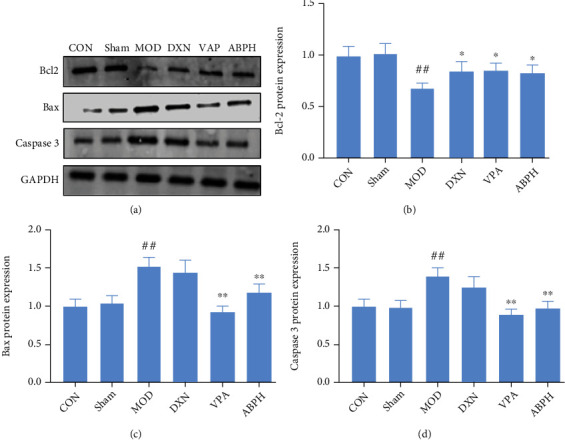
The regulation apoptotic pathway of KA-induced rats with treatment. (a) Western blotting in KA-induced epilepsy rats with treatment; (b) the expression of Bcl2 in KA-induced epilepsy rats with treatment; (c) the expression of Bax in KA-induced epilepsy rats with treatment; (d) the expression of caspase 3 in KA-induced epilepsy rats with treatment.

As shown in Figure 8, KA decreased the expression of Bcl-2 and increased the expression of Bax and caspase 3 (*P* < 0 01). Moreover, ABPH could improve the KA-induced neuronal apoptosis, increased the expression of Bcl-2, and decreased the expression of Bax and caspase 3 (*P* < 0 01).

**Figure 9:**
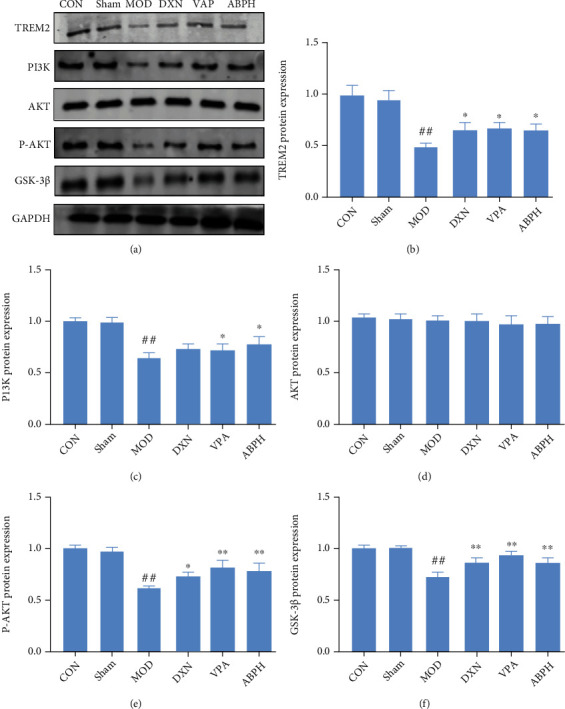
The regulation of oxidative stress pathways of KA-induced rats with treatment. (a) Western blotting in KA-induced epilepsy rats with treatment; (b) the expression of TREM 2 in KA-induced epilepsy rats with treatment; (c) the expression of PI3K in KA-induced epilepsy rats with treatment; (d) the expression of AKT in KA-induced epilepsy rats with treatment; (e) the expression of P-AKT in KA-induced epilepsy rats with treatment; (f) the expression of GSK-3*β* in KA-induced epilepsy rats with treatment.

The expression of TREM2, PI3K, P-Akt, and GSK3*β* was significantly lower in the epilepsy group rats than in the CON and sham group rats, and there was no significant change in Akt expression. Compared with the MOD group, ABPH could decrease the expressions of TREM2, PI3K, P-Akt, and GSK3*β*. Thus, we hypothesized that ABP might activate the PI3K/Akt/GSK-3*β* pathway by regulating TREM2, as shown in Figure 9.

### 3.7. Effect of ABP on PC12 Cells Induced by H_2_O_2_

**Figure 10:**
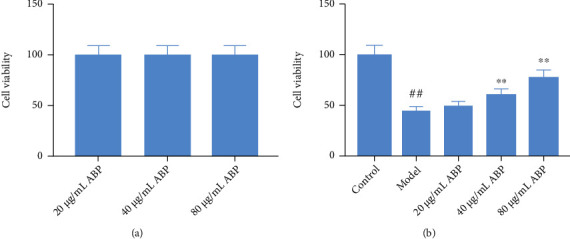
The effect of ABP on PC12 cells induced by H_2_O_2_. (a) The cell viability of different doses of BEO; (b) the effect of different doses of BEO on hippocampal neuron model of epilepsy. Compared with the control group, the difference was significant ^(#^*P* < 0.05); compared with the model group, the difference was significant (^∗^*P* < 0.05).

**Figure 11:**
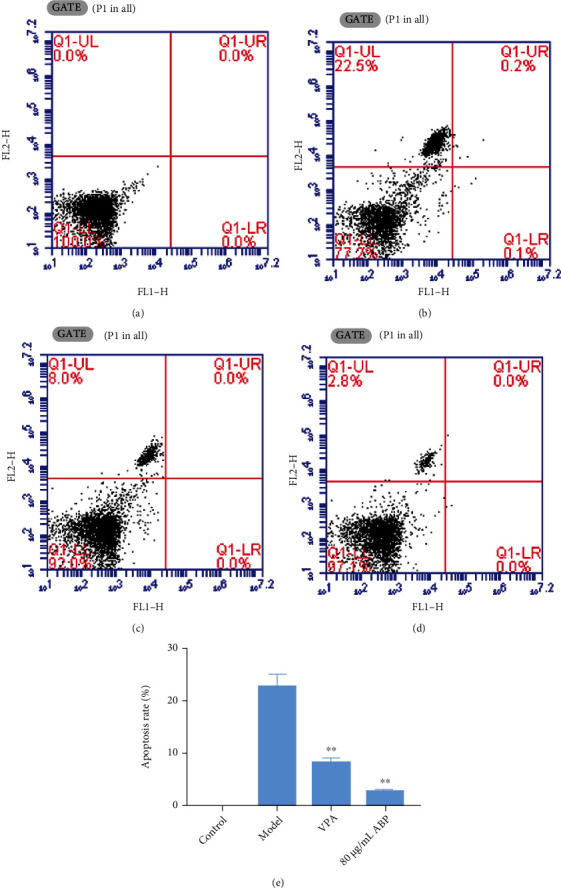
The antiapoptosis effect of ABP on PC12 cells induced by H_2_O_2_. Compared with the model group, the difference was significant (^∗^*P* < 0.05).

The viability of the model group cells was significantly lower than that of the control group cells (*P* < 0.01). The viability of the ABP group cells was higher than that of the model group cells, and the increase in cell viability was more obvious with the increase in ABP concentration (*P* < 0.01) (Figure 10). At the same time, the flow cytometry results also confirmed that ABP could reduce the apoptosis caused by oxidative stress, as shown in Figure 11.

### 3.8. Regulation of the Oxidative Stress Pathways in H_2_O_2_-Induced PC12 Cells

**Figure 12:**
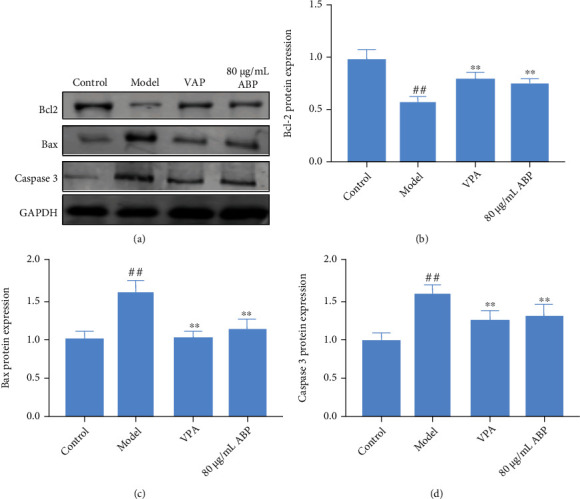
The regulation apoptotic pathway of H_2_O_2_-induced PC12 cells with treatment. (a) Western blotting in H_2_O_2_-induced PC12 cells with treatment; (b) the expression of Bcl2 in H_2_O_2_-induced PC12 cells with treatment; (c) the expression of Bax in H_2_O_2_-induced PC12 cells with treatment; (d) the expression of caspase 3 in H_2_O_2_-induced PC12 cells with treatment.

**Figure 13:**
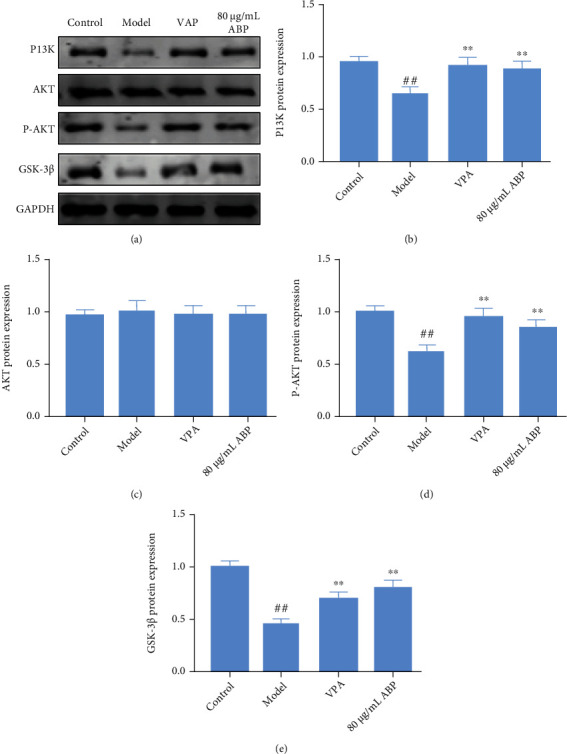
The regulation of oxidative stress pathway of H_2_O_2_-induced PC12 cells with treatment. (a) Western blotting in H_2_O_2_-induced PC12 cells with treatment; (b) the expression of PI3K in H_2_O_2_-induced PC12 cells with treatment; (c) the expression of AKT in H_2_O_2_-induced PC12 cells with treatment; (d) the expression of P-AKT in H_2_O_2_-induced PC12 cells with treatment; (f) the expression of GSK-3*β* in H_2_O_2_-induced PC12 cells with treatment. Compared with the control group, the difference was significant ^(#^*P* < 0.05); Compared with the model group, the difference was significant (^∗^*P* < 0.05).

As shown in Figures 12 and 13, the levels of caspase-3 and Bax proteins in the model cells were upregulated, whereas the expression levels of Bcl-2, PI3K, p-Akt, and GSK-3*β* were downregulated, compared with those in the control cells (*P* < 0 01). The caspase-3 and Bax protein levels were significantly lower in all the ABP-treated (80 *μ*g/mL) cells than in the model cells, whereas the Bcl-2, PI3K, p-Akt, and GSK-3*β* (*P* < 0 05) levels increased following treatment with 80 *μ*g/mL ABP.

## 4. Discussion

Epilepsy is a common chronic neurological disease whose pathogenesis is not completely clarified, and it often requires long-term antiepileptic drug treatment; the treatment of refractory epilepsy is also very difficult. In recent years, there has been increasing evidence of a close link between epileptic seizures and oxidative stress [26]. Polysaccharides from traditional Chinese medicine have attracted extensive attention because of their unique antioxidant ability [12, 27]. In this study, the antiepileptic effects of the ABP were studied (Figures 2–5).

Microinjection of KA in the hippocampus of rats could cause the death of neurons in hippocampal CA1 and CA3 regions [28, 29], dentate gyrus, and other brain regions. Pyramidal cells in the hippocampal CA3 region synapse with mossy fibers and receive excitatory afferents from mossy fibers. Due to the loss of pyramidal cells and mossy cells in the CA3 region, mossy fibers lose their postsynaptic targeting cell connections, resulting in the sprouted mossy fibers to the granulosa cell layer and molecular layer. It could form new excitatory synaptic connections with itself or other granulosa cells, enhance excitatory synaptic transmission of glutamate, and destroy the excitatory and inhibitory balance in the hippocampus, thus causing seizures [30, 31]. Through the analysis of pathology and neurotransmitters, it was found that ABP could improve the neuronal injury in CA1 and CA3 areas and neurotransmitter abnormalities in the hippocampal (Figures 3–5 and 7). And we found that oxidative stress was also an important cause of hippocampal injury [26, 32]. Therefore, the effects of oxidative stress indexes on epileptic rats and ABP on oxidative stress indexes were investigated in the follow-up study.

Free radicals are related to the pathogenesis of many nervous system diseases, such as epilepsy, cerebral ischemia-reperfusion injury, Alzheimer's disease, and Parkinson's disease [26, 32]. SOD can block the chain reaction of free radicals by catalyzing the disproportionation of superoxide anion free radicals to protect cells from oxidative damage. MDA, which is a metabolite produced by the reaction of free radicals with unsaturated fatty acids, is often used to indirectly reflect the changes in free radical metabolism and the degree of cell damage. The determination of the SOD levels is often combined with the determination of the MDA levels to reflect the severity of the free radical attack on cells and the ability to scavenge the oxygen free radicals [33]. In our study, ABP reduced the MDA content and enhanced the activity of SOD in a KA-induced epilepsy rat model (Figure 6). It was speculated that ABP could scavenge hydroxyl radicals and inhibit spontaneous or hydroxyl radical-induced lipid peroxidation. LDH is an important oxidoreductase in the body. Changes in the LDH content could reflect tissue damage to a certain extent. This is a sensitive biochemical index that reflects cell activity and function [34], and its release can be used as a cell damage marker. Through pharmacodynamics and pathological evaluation, we could find that the efficacy of the high dose of ABP significantly decreased epileptic scores and SE% and also improved neuronal damage in hippocampal tissue, but the low doses had no significant effect. And by reviewing the literature [35, 36], our selection is consistent with the report, so only the high dose of ABP was selected for the oxidative stress index detection. In our study, ABPH reduced the MDA content, enhanced the activity of SOD, and increased LDH release in a KA-induced epilepsy rat model (Figure 6). It was speculated that ABP could scavenge hydroxyl radicals and inhibit spontaneous or hydroxyl radical-induced lipid peroxidation.

The apoptosis of neurons after the KA-induced epileptic seizures was observed in the rats throughout the whole process, and the apoptosis of the CA3 region in rats was also obvious. Caspase-3 is a key protein in the apoptosis pathway, and programmed cell death could be caused by the action of cytokines in the Bcl-2 family of proteins, which are important regulatory factors of the mitochondrial pathway. Bcl-2 and Bax are two genes in the Bcl-2 family that are closely related to apoptosis [37]. They are a pair of apoptosis regulatory genes that are functionally opposite to each other and the protein product they express. Bcl-2 protein can inhibit the apoptosis of cells, while Bax protein promotes apoptosis and antagonizes the function of Bcl-2 [38]. As a soluble protein, Bax is mainly located in the cytoplasm or the skeleton of cells. When apoptosis is induced by injury factors, Bax is rapidly transferred to the mitochondria and binds to Bcl-2; the resulting dimer can induce apoptosis. In both rat and cell models, we observed that ABP might improve apoptosis by regulating the expression of caspase 3 and Bcl-2/Bax (Figures 4 and 8).

The excitatory glutamate toxicity increases in the hippocampal tissue during seizures. A large number of oxygen free radicals can promote hippocampal nerve cell apoptosis and even necrosis, and activating the PI3K/Akt signaling pathway can substantially inhibit hippocampal nerve cell apoptosis [39]. In this study, we speculated that ABP activates the PI3K/Akt signaling pathway, which is involved in the apoptosis of hippocampal neurons. The PI3K/Akt signaling pathway is important for maintaining cell apoptosis and growth by regulating the downstream apoptotic proteins and cyclins (Figures 3, 11, and 13). Akt is a target kinase that is downstream of PI3K. When stimulated by intracellular signals, PI3K activates PIP3 production, phosphorylates Akt, and triggers a cascade reaction, thereby regulating cell apoptosis [40]. GSK-3*β* is a subtype of GSK-3, which can phosphorylate multiple transcription factors and proteins and plays an important role in initiating apoptosis [41]. The PI3K/Akt signaling pathway has the most apparent effect on GSK-3*β* [40]. This experiment showed that the levels of the PI3K/P-Akt protein complex and the downstream molecule GSK-3*β* were lower in the MOD group than in the CON group and that ABP modulated the abnormal levels of these proteins. These results suggest that ABP regulates the PI3K/Akt/GSK-3*β* pathway to suppress neuronal apoptosis and are consistent with the cell experiment results (Figures 9 and 13).

Recently, a study has shown that the expression of TREM2 is downregulated in an epileptic hippocampal neuron model, which could repair the damaged central nervous system tissue and further decrease the phagocytosis of apoptotic neurons via the NF-*κ*B pathway [39, 40, 42]. Therefore, we studied the regulation of TREM2 expression in epilepsy via the PI3K/Akt pathway in an epileptic rat model. Importantly, our results show that TREM2 can activate the PI3K/Akt signaling pathway, inhibit the apoptosis and injury of hippocampal neurons, and reduce oxidative stress. In summary, ABP upregulated the expression of TREM2 to suppress the expression of the apoptosis-related proteins Bax and caspase 3, increase the Bcl-2 expression, and activate the PI3K/Akt pathway, which possibly holds therapeutic implications for inhibiting neuronal apoptosis.

At present, this study is limited to the unknown structure of the polysaccharides from the aerial part of *Bupleurum chinense* DC.. Some further characterizations such as sugar linkages and anomeric configuration are highly desirable. Since all properties particularly the biological properties of polysaccharides are controlled by the molecular structure, the detail of structure is essential. Therefore, we will further study the polysaccharide structure of the aerial part of *Bupleurum chinense* DC., to reveal the correlation between the polysaccharide structure and its mechanism of epilepsy.

## 5. Conclusions

**Figure 14:**
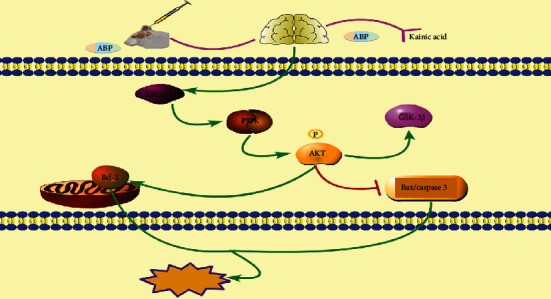
The therapeutic mechanism of ABP treats KA-induced epilepsy.

Polysaccharides from the aerial parts of *B. chinense* DC have an average molecular weight of 1.38 × 10^3^ kDa and are composed of galacturonic acid, galactose, arabinose rhamnose, and mannose. In the present study, we evaluated the antiepileptic effects of ABP on KA-induced epileptic rats, the protective effects of ABP against H_2_O_2_-induced injury in PC12 cells, and the underlying molecular mechanisms. ABP exerted remarkable antiepileptic effects and protective effects against oxidative damage through upregulating TREM2 expression and alleviating hippocampal neuronal injury and oxidative stress. ABP also inhibited oxidative stress by activating the PI3K/Akt/GSK-3*β* pathway (Figure 14). The present study provides insights into the use of the polysaccharides from *B. chinense* DC aerial parts for treating epilepsy.

## Data Availability

The data used to support the findings of this study are included in the article. Further data or information required are available from the corresponding author upon request.

## Abbreviations

ABP: Polysaccharide from the aerial parts of *Bupleurum chinense* DC.
ELISA: Enzyme-linked immunosorbent assay
HE: Haematoxylin-eosin
GAD: Glutamic acid decarboxylase
GIRK: G protein gated inwardly rectifying K channels
DAB: 3,3′-diaminobenzidine tetrahydrochloride
b. w.: Body weight
TFA: Trifluoroacetic acid
SD: Sprague-Dawley
KA: Kainic acid
HPLC: High-performance liquid chromatography
GABA: *γ*-Aminobutyric acid type A
GFAP: Glial fibrillary acidic protein
NMDAR: N-methyl-D-aspartic acid receptor
KBr: Potassium bromide
p. o.: Oral
TCM: Traditional Chinese medicine.
